# Supplementary material for: Ribosome demand links transcriptional bursts to protein expression noise
Source: eLife. 2026 Feb 18;13:RP99322. doi: 10.7554/eLife.99322 (PMC12916104; doi:10.7554/eLife.99322)
Supplement: Supplementary file 1. [file elife-99322-supp1.docx]

Supplementary File 1

List of mathematical functions explored to model ribosome demand

mRNA_prev_ - Number of mRNA molecules at the previous time-point during simulation

mRNA_curr_ - Number of mRNA molecules present at the present time-point during simulation

ribo_prev_  - Number of ribosomes that were bound to mRNA molecules at the previous time-point during simulation

ribo_curr_ - Number of ribosomes that are bound to mRNA molecules at the current time-point during simulation

tlinitr_base_ – The basal translation initiation rate

tlinitr_curr_ - the translation initiation rate at the current time-point during simulation

| **Function number (FN)** | **Function** |
| --- | --- |
| 1 | \| av = $\frac{1+ {mRNA}_{curr}}{1+ {mRNA}_{prev}}$ \| \| --- \| \| kc = 1 + $\frac{5}{(1+ {mRNA}_{prev})}$ \| \| ${tlinitr}_{curr}= {tlinitr}_{base}$ × $\frac{{kc}^{10}}{{kc}^{10} +{av}^{10}}$ \| \|  \| |
| 2 | \| av = $\frac{1+ {mRNA}_{curr}}{1+ {mRNA}_{prev}}$ \| \| --- \| \| kc = 1 + $\frac{6}{(1+ {mRNA}_{prev})}$ \| \| ${tlinitr}_{curr}= {tlinitr}_{base}$ × $\frac{{kc}^{10}}{{kc}^{10} +{av}^{10}}$ \| \|  \| |
| 3 | \| av = $\frac{1+ {mRNA}_{curr}}{1+ {mRNA}_{prev}}$ \| \| --- \| \| kc = 1 + $\frac{10}{(1+ {mRNA}_{prev})}$ \| \| ${tlinitr}_{curr}= {tlinitr}_{base}$ × $\frac{{kc}^{10}}{{kc}^{10} +{av}^{10}}$ \| \|  \| |
| 4 | \| av = $\frac{1+ {mRNA}_{curr}}{1+ {mRNA}_{prev}}$ \| \| --- \| \| kc = [1 + $\frac{10}{(1+ {mRNA}_{prev})}$ ] × $\frac{1}{{(1+ \frac{{ribo}_{curr}}{50})}^{10}}$ \| \| ${tlinitr}_{curr}= {tlinitr}_{base}$ × $\frac{{kc}^{10}}{{kc}^{10} +{av}^{10}}$ \| \|  \| |
| 5 | \| av = $\frac{1+ {ribo}_{curr}}{1+ {ribo}_{prev}}$ \| \| --- \| \| kc = 1 + $\frac{5}{(1+ {ribo}_{prev})}$ \| \| ${tlinitr}_{curr}= {tlinitr}_{base}$ × $\frac{{kc}^{10}}{{kc}^{10} +{av}^{10}}$ \| \|  \| |
| 6 | \| av = $\frac{1+ {ribo}_{curr}}{1+ {ribo}_{prev}}$ \| \| --- \| \| kc = 1 + $\frac{10}{(1+ {ribo}_{prev})}$ \| \| ${tlinitr}_{curr}= {tlinitr}_{base}$ × $\frac{{kc}^{10}}{{kc}^{10} +{av}^{10}}$ \| \|  \| |
| 7 | \| av = $\frac{1+ {ribo}_{curr}}{1+ {ribo}_{prev}}$ \| \| --- \| \| kc = 1 + $\frac{20}{(1+ {ribo}_{prev})}$ \| \| ${tlinitr}_{curr}= {tlinitr}_{base}$ × $\frac{{kc}^{10}}{{kc}^{10} +{av}^{10}}$ \| \|  \| |
| 8 | \| av = $\frac{1+ {ribo}_{curr}}{1+ {ribo}_{prev}}$ \| \| --- \| \| kc = 1 + $\frac{30}{(1+ {ribo}_{prev})}$ \| \| ${tlinitr}_{curr}= {tlinitr}_{base}$ × $\frac{{kc}^{10}}{{kc}^{10} +{av}^{10}}$ \| \|  \| |
| 9 | \| av = $\frac{1+ {ribo}_{curr}}{1+ {ribo}_{prev}}$ \| \| --- \| \| kc = 1 + $\frac{40}{(1+ {ribo}_{prev})}$ \| \| ${tlinitr}_{curr}= {tlinitr}_{base}$ × $\frac{{kc}^{10}}{{kc}^{10} +{av}^{10}}$ \| \|  \| |
| 10 | \| av = $\frac{1+ {ribo}_{curr}}{1+ {ribo}_{prev}}$ \| \| --- \| \| kc = 1 + $\frac{50}{(1+ {ribo}_{prev})}$ \| \| ${tlinitr}_{curr}= {tlinitr}_{base}$ × $\frac{{kc}^{10}}{{kc}^{10} +{av}^{10}}$ \| \|  \| |
| 11 | \| av = $\frac{1+ {mRNA}_{curr}}{1+ {mRNA}_{prev}}$ \| \| --- \| \| ${tlinitr}_{curr}= {tlinitr}_{base}$ × $\frac{1}{av}$ \| \|  \| |
| 12 | \| av = $\frac{1+ {ribo}_{curr}}{1+ {ribo}_{prev}}$ \| \| --- \| \| ${tlinitr}_{curr}= {tlinitr}_{base}$ × $\frac{1}{av}$ \| \|  \| \|  \| |
| 13 | \| av = $\left( \frac{1+ {mRNA}_{curr}}{1+ {mRNA}_{prev}} \right) \times\left( \frac{1+ {ribo}_{curr}}{1+ {ribo}_{prev}} \right)$ \| \| --- \| \| ${tlinitr}_{curr}= {tlinitr}_{base}$ × $\frac{1}{av}$ \| \|  \| |
| 14 | \| av = $\left( \frac{1+ {mRNA}_{curr}}{1+ {mRNA}_{prev}} \right) \times\left( \frac{1+ {ribo}_{curr}}{1+ {ribo}_{prev}} \right)$ \| \| --- \| \| kc = 1 + $\frac{1}{(1+ {{mRNA}_{prev} + ribo}_{prev})}$ \| \| ${tlinitr}_{curr}= {tlinitr}_{base}$ × $\frac{{kc}^{10}}{{kc}^{10} +{av}^{10}}$ \| \|  \| |
| 15 | \| av = $\left( \frac{1+ {mRNA}_{curr}}{1+ {mRNA}_{prev}} \right) \times\left( \frac{1+ {ribo}_{curr}}{1+ {ribo}_{prev}} \right)$ \| \| --- \| \| kc = 1 + $\frac{2}{(1+ {{mRNA}_{prev} + ribo}_{prev})}$ \| \| ${tlinitr}_{curr}= {tlinitr}_{base}$ × $\frac{{kc}^{10}}{{kc}^{10} +{av}^{10}}$ \| \|  \| |
| 16 | \| av = $\left( \frac{1+ {mRNA}_{curr}}{1+ {mRNA}_{prev}} \right) \times\left( \frac{1+ {ribo}_{curr}}{1+ {ribo}_{prev}} \right)$ \| \| --- \| \| kc = 1 + $\frac{5}{(1+ {{mRNA}_{prev} + ribo}_{prev})}$ \| \| ${tlinitr}_{curr}= {tlinitr}_{base}$ × $\frac{{kc}^{10}}{{kc}^{10} +{av}^{10}}$ \| \|  \| |
| 17 | \| av = $\left( \frac{1+ {mRNA}_{curr}}{1+ {mRNA}_{prev}} \right) \times\left( \frac{1+ {ribo}_{curr}}{1+ {ribo}_{prev}} \right)$ \| \| --- \| \| kc = 1 + $\frac{10}{(1+ {{mRNA}_{prev} + ribo}_{prev})}$ \| \| ${tlinitr}_{curr}= {tlinitr}_{base}$ × $\frac{{kc}^{10}}{{kc}^{10} +{av}^{10}}$ \| \|  \| |
| 18 | \| av = $\left( \frac{1+ {mRNA}_{curr}}{1+ {mRNA}_{prev}} \right) \times\left( \frac{1+ {ribo}_{curr}}{1+ {ribo}_{prev}} \right)$ \| \| --- \| \| kc = 1 + $\frac{15}{(1+ {{mRNA}_{prev} + ribo}_{prev})}$ \| \| ${tlinitr}_{curr}= {tlinitr}_{base}$ × $\frac{{kc}^{10}}{{kc}^{10} +{av}^{10}}$ \| \|  \| |
| 19 | \| av = $\left( \frac{1+ {mRNA}_{curr}}{1+ {mRNA}_{prev}} \right) \times\left( \frac{1+ {ribo}_{curr}}{1+ {ribo}_{prev}} \right)$ \| \| --- \| \| kc = 1 + $\frac{25}{(1+ {{mRNA}_{prev} + ribo}_{prev})}$ \| \| ${tlinitr}_{curr}= {tlinitr}_{base}$ × $\frac{{kc}^{10}}{{kc}^{10} +{av}^{10}}$ \| \|  \| |
