# Supplementary material for: Ribosome demand links transcriptional bursts to protein expression noise
Source: eLife. 2026 Feb 18;13:RP99322. doi: 10.7554/eLife.99322 (PMC12916104; doi:10.7554/eLife.99322)
Supplement: Supplementary file 2. [file elife-99322-supp2.docx]

Supplementary File 2

List of primers used

| **Primer name** | **Sequence (5’ to 3’)** |
| --- | --- |
| GFP_for | AGCGGTACCATGTCTAAAGGAGAAGAACTTTTCACTGGAG |
| GFP_rev | AGCGGATCCCTATTTGTATAGTTCATCCATGCCATGTG |
| His3L_for | AGCGAATTCAACACAGTCCTTTCCCGCA |
| His3L_rev | AGCGAGCTCCTTTGCCTTCGTTTATCTTGCCTG |
| His3R_for | AGCGGATCCGTCGACTGACACCGATTATTTAAAG |
| His3R_rev | AGCAAGCTTAATATGAAATGCTTTTCTTGTTGTTCTTAC |
| HIS3MX_for | AGCGGATCCGGCGCGCCACTTCTAAATAAG |
| HIS3MX_rev | AGCAAGCTTGTCGACCAGTATAGCGACCAG |
| RPL35Ap_for | AGCGAGCTCAAATTCTAGAATATGGATCAAATACGCTTG |
| RPL35Ap_rev | AGCGGTACCTTCGAATATACTGTCTCACTGTACC |
| CPA2p_for | AGCGAGCTCCTTTGCGGAATGGTATACTATTTCTTTTC |
| CPA2p_rev | AGCGGTACCTCTTTTCTTCCTGTCTATGTACTGTATTG |
| QCR2p_for | AGCGAGCTCTTGGATCTTGCTCAACAAAAATTTTTC |
| QCR2p_rev | AGCGGTACCCAACGTTCTTCTTTTTTCCTTTTAATAATTTTAAC |
| RPG1p_for | AGCGAGCTCTTATTTCAAATTTTTCATGTCCTTGTATTTTTATTC |
| RPG1p_rev | AGCGGTACCCTTGATGTTTCCTGGGCGTTTG |
| NGFP_TCT2TCG_for | AGCGGTACCATGTCGAAAGGAGAAGAACTTTTCACTGGA |
| NGFP_GGA4GGT_for | AGCGGTACCATGTCTAAAGGTGAAGAACTTTTCACTGGA |
| RPG1-TCT2TCG_rev | GAAAAGTTCGTCTCCCTTAGACATGGTACCCTTGATGTTTCC |
| RPG1-GGA4GGT_rev | GAAAAGTTCCTCTCCTTTAGACATGGTACCCTTGATGTTTCC |
| RPL35A-TCT2TCG_rev | GAAAAGTTCGTCTCCCTTTGACATGGTACCTTCGAATATACTGTC |
| RPL35A-GGA4GGT_rev | GAAAAGTTCCTCTCCTTTTGACATGGTACCTTCGAATATACTGTC |
| CPA2-TCT2TCG_rev | GAAAAGTTCGTCTCCCTTAGACATGGTACCTCTTTTCTTCCTGTC |
| CPA2-GGA4GGT_rev | GAAAAGTTCCTCTCCTTTAGACATGGTACCTCTTTTCTTCCTGTC |
| QCR2-TCT2TCG_rev | GAAAAGTTCGTCTCCCTTAGACATGGTACCCAACGTTCTTC |
| QCR2-GGA4GGT_rev | GAAAAGTTCCTCTCCTTTAGACATGGTACCCAACGTTCTTC |
| NGFP_GAA5GAG_for | AGCGGTACCATGTCTAAAGGAGAGGAACTTTTCACTGGA |
| NGFP_GAA6GAC_for | AGCGGTACCATGTCTAAAGGAGACGAACTTTTCACTGGA |
| NGFP_GAT234GAC_rev | AGCGGATCCCTATTTGTATAGTTCGTCCATGCCATGTGT |
| NGFP_CTA236CTC_rev | AGCGGATCCCTATTTGTAGAGTTCATCCATGCCATGTGT |
| NGFP_AAA238AAG_rev | AGCGGATCCCTACTTGTATAGTTCATCCATGCCATGTGT |
| N-5_for | AGCGGTACCATGTCGAAGGGTGAGGACCTTTTCACTGGA |
| C-5_rev | AGCGGTACCATGTCGAAGGGTGAGGACCTTTTCACTGGA |
| RPG1-GAA5GAG_rev | GAAAAGTTCCTCTCCTTTAGACATGGTACCCTTGATGTTTCC |
| RPG1- GAA6GAC_rev | GAAAAGGTCGTCTCCTTTAGACATGGTACCCTTGATGTTTCC |
| RPG1-N-5_rev | GAAAAGGTCCTCACCCTTCGACATGGTACCCTTGATGTTTCC |
| QCR2- GAA5GAG _rev | GAAAAGTTCCTCTCCTTTAGACATGGTACCCAACGTTCTTC |
| QCR2- GAA6GAC_rev | GAAAAGGTCGTCTCCTTTAGACATGGTACCCAACGTTCTTC |
| QCR2-N-5_rev | GAAAAGGTCCTCACCCTTCGACATGGTACCCAACGTTCTTC |
| CPA2- GAA5GAG _rev | GAAAAGTTCCTCTCCTTTAGACATGGTACCTCTTTTCTTCCTGTC |
| CPA2- GAA6GAC_rev | GAAAAGGTCGTCTCCTTTAGACATGGTACCTCTTTTCTTCCTGTC |
| CPA2-N-5_rev | GAAAAGGTCCTCACCCTTCGACATGGTACCTCTTTTCTTCCTGTC |
| GAT234GAC-HMX_for | GGCATGGACGAACTATACAAATAGGGATCCGGCGCGCCACTTC |
| CTA236CTC-HMX_for | GGCATGGATGAACTCTACAAATAGGGATCCGGCGCGCCACTTC |
| AAA238AAG-HMX_for | GGCATGGATGAACTATACAAGTAGGGATCCGGCGCGCCACTTC |
| C-5-HMX_for | GGCATGGACGACCTCAACAAGTAGGGATCCGGCGCGCCACTTC |
