# Supplementary material for: Ribosome demand links transcriptional bursts to protein expression noise
Source: eLife. 2026 Feb 18;13:RP99322. doi: 10.7554/eLife.99322 (PMC12916104; doi:10.7554/eLife.99322)
Supplement: Supplementary file 3. [file elife-99322-supp3.docx]

**Supplementary File 3**

Fragment size and nucleotide sequence of the parts of the promoter-GFP constructs

| **Fragment** | **Sequence** | **Size (bps)** |
| --- | --- | --- |
| His3L | AACACAGTCCTTTCCCGCAATTTTCTTTTTCTATTACTCTTGGCCTCCTCTAGTACACTCTATATTTTTTTATGCCTCGGTAATGATTTTCATTTTTTTTTTTCCACCTAGCGGATGACTCTTTTTTTTTCTTAGCGATTGGCATTATCACATAATGAATTATACATTATATAAAGTAATGTGATTTCTTCGAAGAATATACTAAAAAATGAGCAGGCAAGATAAACGAAGGCAAAG | 237 |
| RPL35A  promoter | AAATTCTAGAATATGGATCAAATACGCTTGTATAAACTAAATGAAACATAAAGATTAAGAACTTAAGAGGCCAACGTCGATGGATTTATTGACGATCACCAGCCAACACATATAGATTTTAGTGTAAAAGCAATAAAAACCAAGATAATAAAATAAAAAAATACTGAAGAAGCCTAACTAGTATAAACTACTTTAACTAATAATGGCAATTTGATATAGAAACAAAGAAACATGATATATTTAGGATATTATACAACGCATTTTCATTTGTTTTACAGCACCCCTGCGTGAATCATATATTGACGTTTCGCTCTCAGGTCCACCGTGTTCTCAAAAGATACTTTTAAAACCTAAAACACACGAAATCATATTATGATAATTCAGAATGATAGTGTGGTACTGTGTCAATTGACTGTTCAAGACTGAAGAGGATCTTTGATTTGTTGTTACTCAACAAATAATCTTCACGAAAACTTTCTCAATCTGGGGACTGTATTAATCTCAGACCCATACATATCTACACCCATAACTTTTTACATTTAATTTTTTATCACATAATAGGTAGCTTAAATTGTAAAGTCGCAAAAAAAAATGGCAGCGCAGCCTCTCCGGGTGAACCCCACGACAACTTACCTGGCACTCCATGCACTAACGGGCGGGTTTGGGCAGGATTCCAGCATCAATTTTGCAAAATTCACACCTGAGTAATTCATATATGTAATATAATGTTAAGCATACGCTGTCGATTAGCACTATTATTGACCGTAGAATAGGTACAGTGAGACAGTATATTCGAA | 797 |
| RPG1 promoter | TTATTTCAAATTTTTCATGTCCTTGTATTTTTATTCTTTATCCCTTCCAATCAGAAAGGATCTAGTGAACAAGTTCTTTCCTCTATGGTATATATTTTAGTGATAAATTTTATAAAATTATCAAAACCAAGGCATCCTTTCCTTTTATTCTGTCATTGGAATCTGCCTGTCATAATTATCACCCACCGGGTAAAGATGATAATTTTTCAGTCGCTTTGCCCGAGAAGCTTTGCCAGGTGAAAAATTTTTCTTGGTGAACTAAATCGAAAGTAGATATACTTACAACTATAGAGAGAGTTCCAAAATAAACCATACAAACGCCCAGGAAACATCAAG | 336 |
| CPA2 promoter | CTTTGCGGAATGGTATACTATTTCTTTTCCCTCTTTTTAGCACTATTACACCCCGCCCACAAAATAAAAATAATAACGACGACCTAATCTCACCAAGTGACCCTTGTAAAACCTCCTTTTCTTTATAATGTTTCTTTTCTTACTAATATTTGGTACATTTAGGGTAGTGATAAAAGAATGGCAACATTGTTATTATTGTGAAAAATGAGGAAAATAGAAAATCAGAAACCCTAAAAAGTGATTTTACCCTATCAGAAATATTCAAATGTCCTAATTAAAAATAGTAAATCCCCTAAACATTCAGATTGTAAACTAGGGTTGAGAAAATGACTCATCCACCACTGTCTTCTTTCCTGCGGCATTCTATAGATTATTGTGAATGACTCTTATTGATGAGATGGCAATAACTTTTGAATATCAGAGATAGGAACCTCCATGTCGTAACGATTGTGTCACCTTGAGTAAGCATCGAGAAAATCCAATCTTTTTTTTTCCGTCATAAGCATTTCTGCCATGCTATTTGTATATATATAATTACTAATACGTCTTCTATAGTATGCCTTATCTCTTTTTTGAAGCGCTATTTAAGTTTAAGCATCGAAAAACTAACATCTATAGTTAAAATTAGTTCTATAAAGGAAGAGCAATACAGTACATAGACAGGAAGAAAAGA | 673 |
| QCR2  promoter | TTGGATCTTGCTCAACAAAAATTTTTCCCTATTCCTGTGTCAGCAATTCTCTATTGTTCTCAATATTTCTTCCACTATTATTATTTTGATCCAAAATTATTTTTTTCCTTCAATGCGATGAGCTTTTGAAAAATTTCTGATCATTCCCAACGAACCAATAGAAGGCCCGCCCCGTCTTATATCCGTTAGCCTACCAAATATATATATAAAGAACAAGGGCCTTTCCTCAGAGCGTTTGCTGACGAAGTTTTAGAAGTTAATAAGGTTTTTAACAGCAGTGTGCTCGAACGATTAGGACGGGAGAGTTAAAATTATTAAAAGGAAAAAAGAAGAACGTTG | 339 |
| GFP | ATGTCTAAAGGAGAAGAACTTTTCACTGGAGTTGTCCCAATTCTTGTTGAATTAGATGGTGATGTTAATGGGCACAAATTTTCTGTCAGTGGAGAGGGTGAAGGTGATGCAACATACGGAAAACTTACCCTTAAATTTATTTGCACTACTGGAAAACTACCTGTTCCATGGCCAACACTTGTCACTACTTTTACGTATGGTGTTCAATGCTTTTCAAGATACCCAGATCATATGAAACGGCATGACTTTTTCAAGAGTGCCATGCCCGAAGGTTATGTACAGGAAAGAACTATATTTTTCAAAGATGACGGGAACTACAAGACACGTGCTGAAGTCAAGTTTGAAGGTGATACCCTTGTTAATAGAATCGAGTTAAAAGGTATTGATTTTAAAGAAGATGGAAACATTCTTGGACACAAATTGGAATACAACTATAACTCACACAATGTATACATCATGGCAGACAAACAAAAGAATGGAATCAAAGTTAACTTCAAAATTAGACACAACATTGAAGATGGAAGCGTTCAACTAGCAGACCATTATCAACAAAATACTCCAATTGGCGATGGCCCTGTCCTTTTACCAGACAACCATTACCTGTCCACACAATCTGCCCTTTCGAAAGATCCCAACGAAAAGAGAGACCACATGGTCCTTCTTGAGTTTGTAACAGCTGCTGGGATTACACATGGCATGGATGAACTATACAAATAG | 711 |
| HIS3MX6 | GGCGCGCCACTTCTAAATAAGCGAATTTCTTATGATTTATGATTTTTATTATTAAATAAGTTATAAAAAAAATAAGTGTATACAAATTTTAAAGTGACTCTTAGGTTTTAAAACGAAAATTCTTATTCTTGAGTAACTCTTTCCTGTAGGTCAGGTTGCTTTCTCAGGTATAGCATGAGGTCGCTCTTATTGACCACACCTCTACCGGCAGATCTGTTTAGCTTGCCTCGTCCCCGCCGGGTCACCCGGCCAGCGACATGGAGGCCCAGAATACCCTCCTTGACAGTCTTGACGTGCGCAGCTCAGGGGCATGATGTGACTGTCGCCCGTACATTTAGCCCATACATCCCCATGTATAATCATTTGCATCCATACATTTTGATGGCCGCACGGCGCGAAGCAAAAATTACGGCTCCTCGCTGCAGACCTGCGAGCAGGGAAACGCTCCCCTCACAGACGCGTTGAATTGTCCCCACGCCGCGCCCCTGTAGAGAAATATAAAAGGTTAGGATTTGCCACTGAGGTTCTTCTTTCATATACTTCCTTTTAAAATCTTGCTAGGATACAGTTCTCACATCACATCCGAACATAAACAACCATGGGTAGGAGGGCTTTTGTAGAAAGAAATACGAACGAAACGAAAATCAGCGTTGCCATCGCTTTGGACAAAGCTCCCTTACCTGAAGAGTCGAATTTTATTGATGAACTTATAACTTCCAAGCATGCAAACCAAAAGGGAGAACAAGTAATCCAAGTAGACACGGGAATTGGATTCTTGGATCACATGTATCATGCACTGGCTAAACATGCAGGCTGGAGCTTACGACTTTACTCAAGAGGTGATTTAATCATCGATGATCATCACACTGCAGAAGATACTGCTATTGCACTTGGTATTGCATTCAAGCAGGCTATGGGTAACTTTGCCGGCGTTAAAAGATTTGGACATGCTTATTGTCCACTTGACGAAGCTCTTTCTAGAAGCGTAGTTGACTTGTCGGGACGGCCCTATGCTGTTATCGATTTGGGATTAAAGCGTGAAAAGGTTGGGGAATTGTCCTGTGAAATGATCCCTCACTTACTATATTCCTTTTCGGTAGCAGCTGGAATTACTTTGCATGTTACCTGCTTATATGGTAGTAATGACCATCATCGTGCTGAAAGCGCTTTTAAATCTCTGGCTGTTGCCATGCGCGCGGCTACTAGTCTTACTGGAAGTTCTGAAGTCCCAAGCACGAAGGGAGTGTTGTAAAGGATACTGACAATAAAAAGATTCTTGTTTTCAAGAACTTGTCATTTGTATAGTTTTTTTATATTGTAGTTGTTCTATTTTAATCAAATGTTAGCGTGATTTATATTTTTTTTCGCCTCGACATCATCTGCCCAGATGCGAAGTTAAGTGCGCAGAAAGTAATATCATGCGTCAATCGTATGTGAATGCTGGTCGCTATACTG | 1455 |
| His3R | TGACACCGATTATTTAAAGCTGCAGCATACGATATATATACATGTGTATATATGTATACCTATGAATGTCAGTAAGTATGTATACGAACAGTATGATACTGAAGATGACAAGGTAATGCATCATTCTATACGTGTCATTCTGAACGAGGCGCGCTTTCCTTTTTTCTTTTTGCTTTTTCTTTTTTTTTCTCTTGAACTCGAGAAAAAAAATATAAAAGAGATGGAGGAACGGGAAAAAGTTAGTTGTGGTGATAGGTGGCAAGTGGTATTCCGTAAGAACAACAAGAAAAGCATTTCATATT | 302 |
